# MyD88-Dependent Pathways in Leukocytes Affect the Retina in Diabetes

**DOI:** 10.1371/journal.pone.0068871

**Published:** 2013-07-11

**Authors:** Jie Tang, Chieh Allen Lee, Yunpeng Du, Yan Sun, Eric Pearlman, Nader Sheibani, Timothy S. Kern

**Affiliations:** 1 Department of Medicine, Case Western Reserve University, Cleveland, Ohio, United States of America; 2 Department of Ophthalmology and Visual Science, Case Western Reserve University, Cleveland, Ohio, United States of America; 3 Department of Ophthalmology and Visual Science, University of Wisconsin, Madison, Wisconsin, United States of America; 4 Stokes Veterans Administration Medical Center, Cleveland, Ohio, United States of America; French National Centre for Scientific Research, France

## Abstract

**Background:**

Previous studies by us and other have provided evidence that leukocytes play a critical role in the development of diabetic retinopathy, suggesting a possible role of the innate immune system in development of the retinopathy. Since MyD88 is a convergence point for signaling pathways of the innate immune system (including Toll-Like Receptors (TLRs) and interleukin-1ß (IL-1ß)), the purpose of this study was to assess the role of MyD88 and its dependent pathways on abnormalities that develop in retina and white blood cells related to diabetic retinopathy.

**Methods:**

C57BL/6J mice were made diabetic with streptozotocin. Chimeric mice were generated in which MyD88-dependent pathways were deleted from bone marrow-derived only. Mice were sacrificed at 2 mos of diabetes for assessment of, leukostasis, albumin accumulation in neural retina, leukocyte-mediated killing of retinal endothelial cells, and cytokine/chemokine generation by retinas of diabetic mice in response to TLR agonists,

**Results:**

IL-6 and CXCL1 were generated in retinas from diabetic (but not nondiabetic mice) following incubation with Pam3CysK/TLR2, but incubation with other TLR ligands or IL-1ß did not induce cytokine production in retinas from nondiabetic or diabetic mice. Diabetes-induced abnormalities (leukostasis, ICAM-1 expression on the luminal surface of the vascular endothelium, retinal superoxide generation) were significantly inhibited by removing either MyD88 or the signaling pathways regulated by it (TLRs 2 and 4, and IL-1ß) from bone marrow-derived cells only. Leukocyte-mediated killing of endothelial cells tended to be decreased in the marrow-derived cells lacking TLR2/4, but the killing was significantly exacerbated if the marrow cells lacked MyD88 or the receptor for IL-1ß (IL-1ßr).

**Conclusions:**

MyD88-dependent pathways play an important role in the development of diabetes-induced inflammation in the retina, and inhibition of MyD88 might be a novel target to inhibit early abnormalities of diabetic retinopathy and other complications of diabetes.

## Introduction

Diabetes causes a variety of physiologic and molecular abnormalities in the retina that have been grouped together as indicating a local inflammation [1], including increases in expression or activity of NF-ĸß, IL-1ß, TNFα, RAGE, iNOS, ICAM-1, and p38 MAPK, as well as increases in leukostasis and accumulation of albumin in the neural retina. These pro-inflammatory changes are important in the pathogenesis of the retinopathy, because inhibiting them blocks the development of vascular lesions of the retinopathy in animals [1–4]. Moreover, leukocytes play a critical role in the pathogenesis of this vascular pathophysiology and histopathology, because deletion of single inflammatory proteins solely from marrow-derived cells significantly inhibits development of the retinal histopathology characteristic of early diabetic retinopathy [5,6].

Inflammatory processes are among the means by which the innate immune system rapidly protects itself after exposure to an antigen or microorganism. Recognition of pathogen recognition molecular patterns (PAMPs) or endogenous Danger Associated Molecular Pathogens (DAMPs) by the innate immune system is mediated by specific binding of a pathogen to pattern recognition receptors, such as Receptor for Advanced Glycation End products (RAGE) [7] and TLRs. MyD88 is a convergence point for signaling from many TLRs (including TLRs 2 and 4) and from IL-1ß. Activation of MyD88 results in nuclear translocation of the transcription factor, NF-ĸß, and this transcription factor regulates expression of chemotactic and pro-inflammatory cytokines and other markers of inflammation, including iNOS [8].

The potential contributions of TLRs and MyD88-dependent processes in development of the molecular and physiological abnormalities that contribute to diabetic retinopathy have not been previously studied. Since white blood cells play causal roles in the development of diabetic retinopathy [5,6], the present study investigates MyD88-dependent processes (involving TLR2/TLR4 and IL-1ß) in leukocytes from diabetic mice, and how these changes affect leukocyte-endothelial interactions in the retina in diabetes.

## Methods

### Animals

Wild-type (WT) C57BL/6J mice were purchased from Jackson Laboratories (Bar Harbor, ME). TLR^-/-^ and MyD88^-/-^ mice (on a C57Bl/6J background) were obtained with permission from Dr. S. Akira, (Research Institute for Microbial Diseases, Osaka University, Japan), and mice deficient in the IL-1ß receptor (IL-1βr^-/-^) mice were obtained from Dr. Y. Iwakura University of Tokyo, Japan.

When the C57BL/6 mice were 20-25 g body weight (about 2 mos old), they were randomly assigned to remain nondiabetic or be made experimentally diabetic. All animal experiments were in accordance with the guidelines for treatment of animals in research outlined by the Association for Research in Vision and Ophthalmology. Diabetes was induced by five sequential daily intraperitoneal injections of a freshly prepared solution of streptozotocin in citrate buffer (pH 4.5) at 60 mg/kg body weight. Insulin was given as needed to achieve slow weight gain without preventing hyperglycemia and glucosuria (typically 0–0.2 units of NPH insulin subcutaneously, 0–3 times per week). The animals remained insulin deficient but not catabolic. The animals had free access to both food and water and were maintained under a 14 h on/10 h off light cycle. Food consumption and body weight were measured weekly. Glycated hemoglobin was measured to estimate the average level of hyperglycemia (Variant kit; Bio-Rad, Hercules, CA). Two weeks after the induction of diabetes, the animals were made chimeric using bone marrow from wildtype, IL1β^-/-^, TLR2/4^-/-^ and MyD88^-/-^ mice (described below). Preliminary studies of each of these mutant strains showed no effect on extent of vascularization or capillary cell density in nondiabetic animals (n=2-3 per group)

Retinas and bone marrow were harvested from all animals at 2 months’ duration of diabetes for physiology measurements and co-culture.

### Generation of chimeric mice

Chimeric animals were generated using methods previously reported by us [5], such that only their bone marrow-derived cells lacked both TLRs 2 and 4 (TLR 2/4^-/-^→ WT), or MyD88 (MyD88^-/-^ →WT) or IL-1βr (IL-1βr^-/-^→WT). (The letters on the left of the arrow designate the genotype of the marrow donor, and the letters following the arrow designate the host genotype.) Control animals in which marrow from WT mice was transplanted back into WT animals also were generated (WT→WT). To make chimeras, WT mice (diabetic or nondiabetic) were irradiated to kill endogenous bone marrow. These mice were treated with two doses (600 rad each; 3 hours apart) of whole-body irradiation from cesium [5]. Immediately following the second dose, recipient mice were injected by the tail vein with 200 µl of DMEM containing 3 x 10^6^ bone marrow cells. These myeloid-derived cells were collected from normal animals (WT) or animals lacking the IL-1ß receptor or TLRs 2 and 4 (TLR 2/4^-/-^), or MyD88 (MyD88^-/-^). To collect the marrow, femurs, humerus and tibias were removed from anesthetized animals, and cleaned of muscle. Both ends of the bones were cut off, and the bone marrow was removed from the shafts by centrifugation (10,000 rpm for 30 s). The pellet was resuspended in sterile buffer (erythrocyte lysis buffer) for 2–3 min, centrifuged, and washed in sterile DMEM, prior to injection into the recipient animals.

### Quantitative measurement of leukostasis

The number of leukocytes adherent to the retinal vasculature was determined at 2 months of diabetes in each group. After anesthesia (ketaset:xylazine; 5:1), the chest cavity of mice was carefully opened and a cardiac catheterization was done in the left ventricle with a 6-gauge perfusion cannula. The right atrium was opened with a scissors to allow outflow. With the heart providing the motive force, the mouse was perfused with PBS to clear erythrocytes and other parts of blood, then fluorescein-coupled Concanavalin A lectin (20 ì μg/ml in PBS; Vector Laboratories, Burlingame, CA) was infused as previously described [9–11]. PBS was perfused again for another 1 min to remove excess Concanavalin A. A flat-mount of retina was prepared, and the brightly fluorescent leukocytes within the entire retinal vasculature was counted.

### ICAM-1 expression on the retinal vasculature

We conjugated anti-ICAM-1 antibody to fluorescent microspheres, injected the labeled beads into the circulation, and let them circulate for 30 min. After perfusing, we then counted the number of beads retained to endothelial surface of the retinal vasculature, and compared the number in diabetics to the number in nondiabetic animals. To conjugate the anti-ICAM-1 (1:1000 dilution, Proteintech Group, Inc., Chicago IL) to 2.0 µm fluorescent beads (Duke Scientific Corp, Palo Alto, CA; prepared as per manufacturer’s instructions), we mixed the ingredients (400µl antibody/10^9^ microspheres). Anti-ICAM-1 antibody was diluted to 0.01mg/ml in 1% BSA, and added to 1x10^7^ microspheres to get a final concentration of 0.01mg/ml. We injected 250 µl of antibody solution containing 1x10^7^ microspheres by tail vein and allowed them to circulate for 30 mins.

### Superoxide measurement

Fresh retinas were analyzed for superoxide production as previously described [9,10,12]. Briefly, retinas were placed in 0.2 ml Krebs/HEPES buffer and allowed to equilibrate in the dark at 37°C under 95% O_2_/5% CO_2_ conditions for 30 min. 0.5 mM lucigenin (Sigma Chemical Company, St. Louis, MO) was added to each tube, and the photon emission was detected via luminometer (Analytical Luminescence Laboratory, San Diego, CA). Retinal protein was quantified per sample (Bio-Rad) and the luminescence was expressed per milligram protein.

### Endothelial co-culture with marrow-derived cells

Immortalized mouse retinal endothelial cells (mREC) [13] were grown in control medium (DMEM with 5 mM glucose) containing 10% serum. The serum concentration reduced to 2% at the time that cells were placed either in 5 mM glucose or high glucose (25 mM). Media was changed every other day for 4 days. When the cells reached 80% confluence (500,000 cells), freshly isolated bone marrow cells (100,000 cells) were added and incubated for 24 additional hrs. Marrow cells isolated from nondiabetic animals were co-cultured with mREC incubated in media containing 5 mM glucose as a normal control, and marrow cells from diabetic animals were co-cultured with mREC incubated in 25 mM glucose for the diabetic control. After 24 hrs, the bone marrow cells were carefully removed from mREC by washing with PBS, and viability of the retinal endothelial cells was measured by the trypan blue extrusion method with a haemocytometer. Cell death was expressed as the percentage of endothelial cells that stained blue with dye. Approximately 200-400 cells were counted in each sample. The experiments were repeated two times with similar results.

### Albumin western blot

At 2 months of diabetes, anesthetized animals were perfused with PBS, and the quality of removal of blood from the retinal vasculature was confirmed visually. Retinal homogenates were separated by SDS-PAGE, electroblotted onto PVDF membrane (Bio-Rad), and transferred to nitrocellulose membrane. Membranes were blocked in Tris-buffered saline containing 0.02% Tween 20 and 5% nonfat milk, washed, and incubated with anti-mouse albumin (1; 1000; Bethyl Laboratories, Inc, Montgomery, TX) for 2 hrs, and then stained with respective horseradish peroxidase coupled secondary antibody (Bio-Rad Laboratories, Inc, Hercules, CA) at a dilution of 1:3000 for 1 hr. After extensive washing, staining was visualized by enhanced chemiluminescence (ECL, Santa Cruz Biotechnology, Santa Cruz, CA). The protein levels were quantitated relative to β-actin (1:3000 dilution, Abcam, Inc., Cambridge, MA) in the same samples. Results are expressed as a percent of values detected in the nondiabetic controls. Protein was quantified with the Bio-Rad protein assay (Bio-Rad Laboratories, Inc, Hercules, CA).

### Retinal incubations for Toll-like receptors (TLR) and cytokine ELISA

Eyes were collected from mice diabetic 2 mos, as well as from age-matched nondiabetic controls. The anterior globe and lens were removed, and the retinas carefully separated from the retinal pigment epithelium with a microspatula. Retinas were incubated in RPMI (with 10% fbs) and stimuli for 6 hrs. Glucose concentration in the media was 5mM for retinas from nondiabetic animals and 30 mM for retinas from diabetic mice. TLR ligands were all purchased from InVivogen, including Pam3CysK (TLR2), PolyI:C (TLR3), LPS (TLR4), flagellin (TLR5) and CpG DNA (TLR9). Supernatants from stimulated retinas were examined for CXCL1 (KC) and IL-6 by 2-site ELISA according to the manufacturer’s directions (BD Biosciences). After removal of the retina, media was collected for cytokine analysis.

### Statistical analysis

All results are expressed as the means ±SD. Statistical analysis was performed using ANOVA followed by Fischer’s post-hoc test. Differences were considered statistically significant when p≤0.05.

## Results

### Animals

All diabetic mice were hyperglycemic, and failed to gain weight at normal rate (Table 1). The degree of hyperglycemia, as denoted by glycated hemoglobin, did not vary among diabetic mice, and glycated hemoglobin levels likewise were greater than normal (2.9% ± 0.1) in all diabetic groups (10.2% ± 1.0, 10.0 ± 1.1, 9.2 ± 1.1, and 10.5 ± 1.1, and 10.6 ± 1.2 respectively). Irradiation had no significant effect on body weight or degree of hyperglycemia.

**Table 1:** Body weight and blood glucose in nondiabetic (N) and diabetic (D) mice before and after irradiation to generate chimeras.

	n	Body weight (g)			Blood glucose (mg/dl)	
		Before	After		Before	After
N	13	24 ±2	34 ±2		140 ±12	142 ±12
D	13	25 ±1	24 ±1		393 ±63	465 ±41
N WT→WT	12	23 ±1	29 ±3		144 ±13	139 ±20
D WT→WT	13	23 ±1	24 ±2		392 ±42	381 ±80
N IL-1ßr^-/-^→WT	14	23 ±1	28 ±2		141 ±19	146 ±29
D IL-1ßr^-/-^→WT	10	23 ±2	22 ±2		414 ±43	478 ±83
N TLR2/4^-/-^→WT	15	23 ±2	29 ±2		147 ±24	134 ±19
D TLR2/4^-/-^→WT	15	23 ±1	21 ±3		414 ±50	483 ±63
N MyD88^-/-^→WT	6	23 ±1	28 ±1		150 ±17	147 ±23
D MyD88^-/-^→WT	6	23 ±2	23 ±1		382 ±64	479 ±58

### Diabetes-induced leukostasis

**Figure 1:**
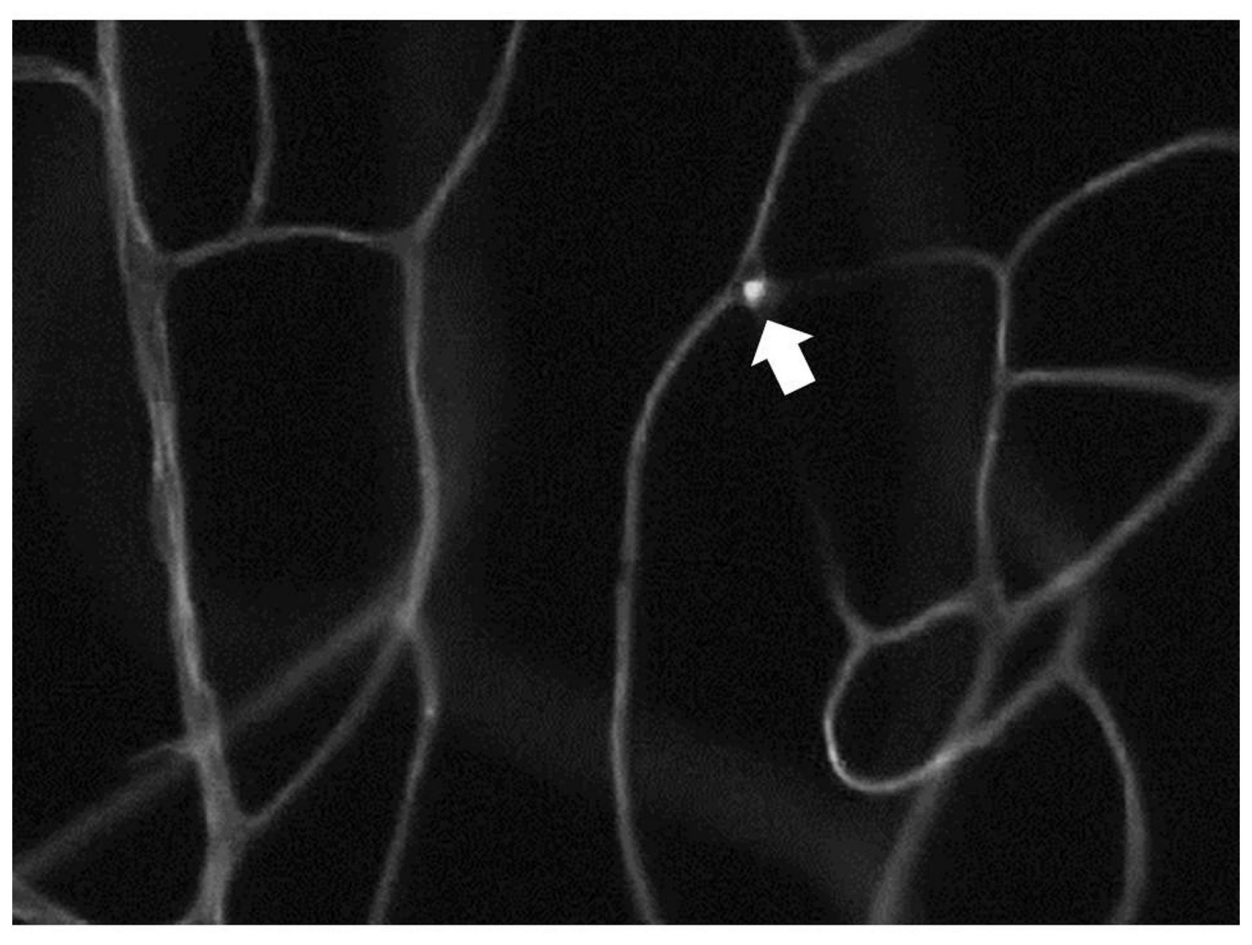
Photomicrograph indicating trapped leukiocytes within the retinal vasculature (leukostasis). The number of these trapped leukocytes is summarized in Figure 2.

**Figure 2:**
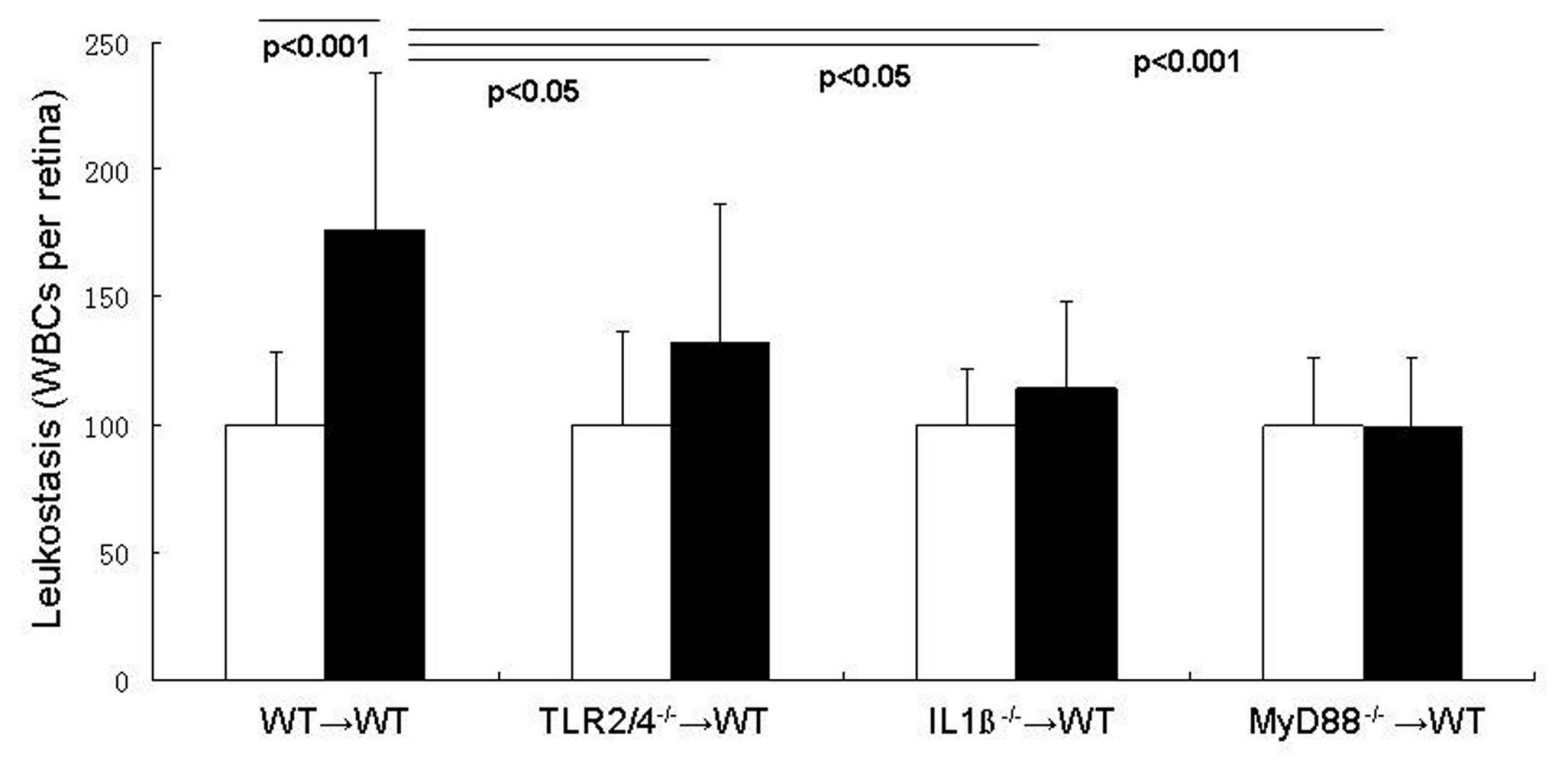
Adherence of leukocytes to the endothelia of the retinal vascular wall (leukostasis) is increased in diabetes, and occurs via MyD88-dependent signalling pathways. Wildtype leukocytes from diabetic mice attached to the vessel wall in significantly greater numbers than did leukocytes from nondiabetic wildtypes, whereas leukocytes lacking either MyD88, TLR2/4 or IL-1βr underwent less diabetes-induced leukostasis than wildtype diabetic controls. Photomicrograph is a representative picture of a diabetic wildtyype animal. n=5 per group; representative of two repeat experiments.

Increased adherence of leukocytes to the vascular wall is an important step in the inflammatory process, and diabetes is known to significantly increase leukocyte adherence to arteries, veins and capillaries in the retina. To investigate the roles of leukocyte TLR2/4, IL-1β, and MyD88 in diabetes-enhanced leukostasis, chimeric mice were made in which these signaling molecules were deleted from bone marrow-derived cells only. Diabetes significantly increased leukostasis in WT mice, and deletion of MyD88 from marrow-derived cells totally inhibited the diabetes-induced increase (the number of leukocytes adhering to retinal vessels in MyD88^-/-^→WT diabetic mice was not significantly different from that in nondiabetic mice) (Figures 1 and 2). In order to determine whether this beneficial effect was initiated by signaling from TLR2/4 or IL-1β, we studied also chimeric mice in which TLR2/4 or IL-1βr were deleted from bone marrow-derived cells only. Deletion of TLR2/4 or IL-1βr from marrow-derived cells partially inhibited the diabetes-induced increase in leukostasis (by 56% or 75% (both P<0.05)). These results demonstrate that both TLR2/4 and IL-1β signaling pathways in leukocytes contribute to the diabetes-induced increase in leukostasis via MyD88.

#### Endothelial ICAM-1 expression

**Figure 3:**
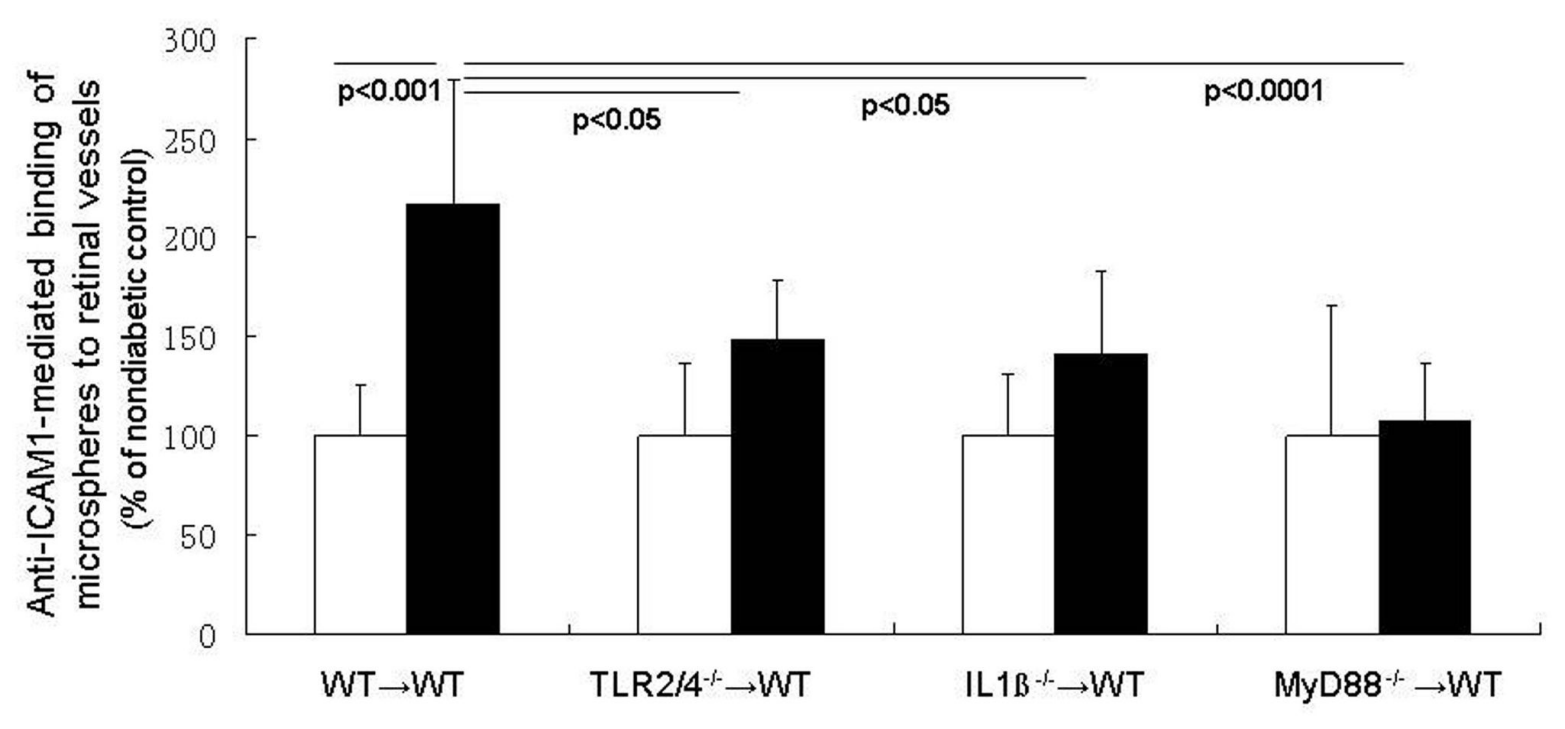
MyD88 pathways in leukocytes influence expression of ICAM-1 on retinal vascular endothelium. ICAM-1 expression is estimated based on the extent of binding of anti-ICAM-1-conjugated microspheres to the retinal vasculature of diabetic and nondiabetic mice. Diabetes in wildtype chimeras caused a significant increase in the number of microspheres binding to the vessels compared to that in nondiabetic wildtype chimeras. ICAM-1 expression in retinal vessels was significantly inhibited in diabetic chimeras lacking either MyD88, TLR2/4, or IL-1βr in their leukocytes. n= 4 per group; representative of two repeat experiments.

In order for leukostasis to occur, leukocytes circulating in the blood bind to adhesion molecules such as ICAM-1 on the endothelial surface. Although signaling pathways within endothelial cells obviously might increase endothelial expression of ICAM-1, we present evidence that marrow-derived cells contribute to the ICAM-1 upregulation on endothelial cells in diabetes. Quantitation of the number of anti-ICAM-1 labeled fluorescent microspheres binding to the luminal surface of the retinal endothelium showed that diabetes significantly increased ICAM-1 expression on retinal vessels in WT animals (WT→WT) two-fold (Figure 3). Deletion of MyD88 from only marrow-derived cells totally inhibited the endothelial ICAM-1 induction in retinas from diabetic animals, and signaling through both TLR2/4 and IL-1β contributed partially to altered regulation of ICAM-1 in diabetes.

### Retinal superoxide generation in diabetes

**Figure 4:**
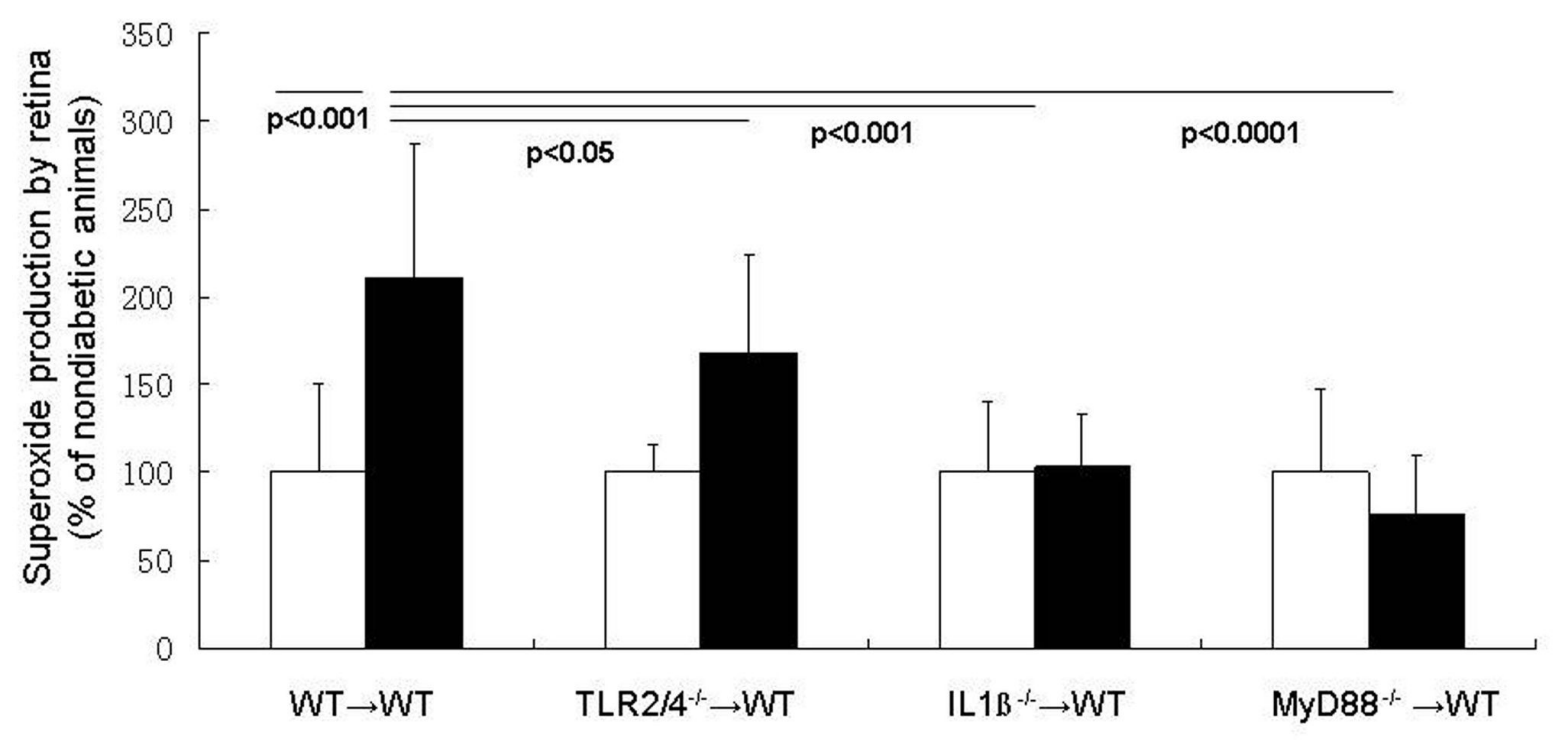
MyD88 pathways in leukocytes regulate diabetes-induced generation of superoxide by the retina. Diabetes caused a doubling of superoxide production by retina in wildype mice, whereas deletion of MyD88, IL-1βr or TLR2/4 only from marrow-derived cells significantly inhibited the diabetes-induced increase in superoxide generation by the retina. n=5-8 per group.

As reported previously by us [9,12,14–16], retinas isolated from wildtype mice diabetic for 2 mos (WT or WT→WT controls) release significantly greater than normal amounts of superoxide (P< 0.001; Figure 4). This diabetes-induced increase in retinal superoxide production was totally inhibited by deleting either MyD88 or IL-1ßr from bone marrow-derived cells only (Figure 4). Deletion of TLR 2/4 from bone marrow-derived cells had only a partial (although statistically significant) effect on the diabetes-induced release of superoxide from the retina.

### Leukocyte-mediated killing of retinal endothelial cells

**Figure 5:**
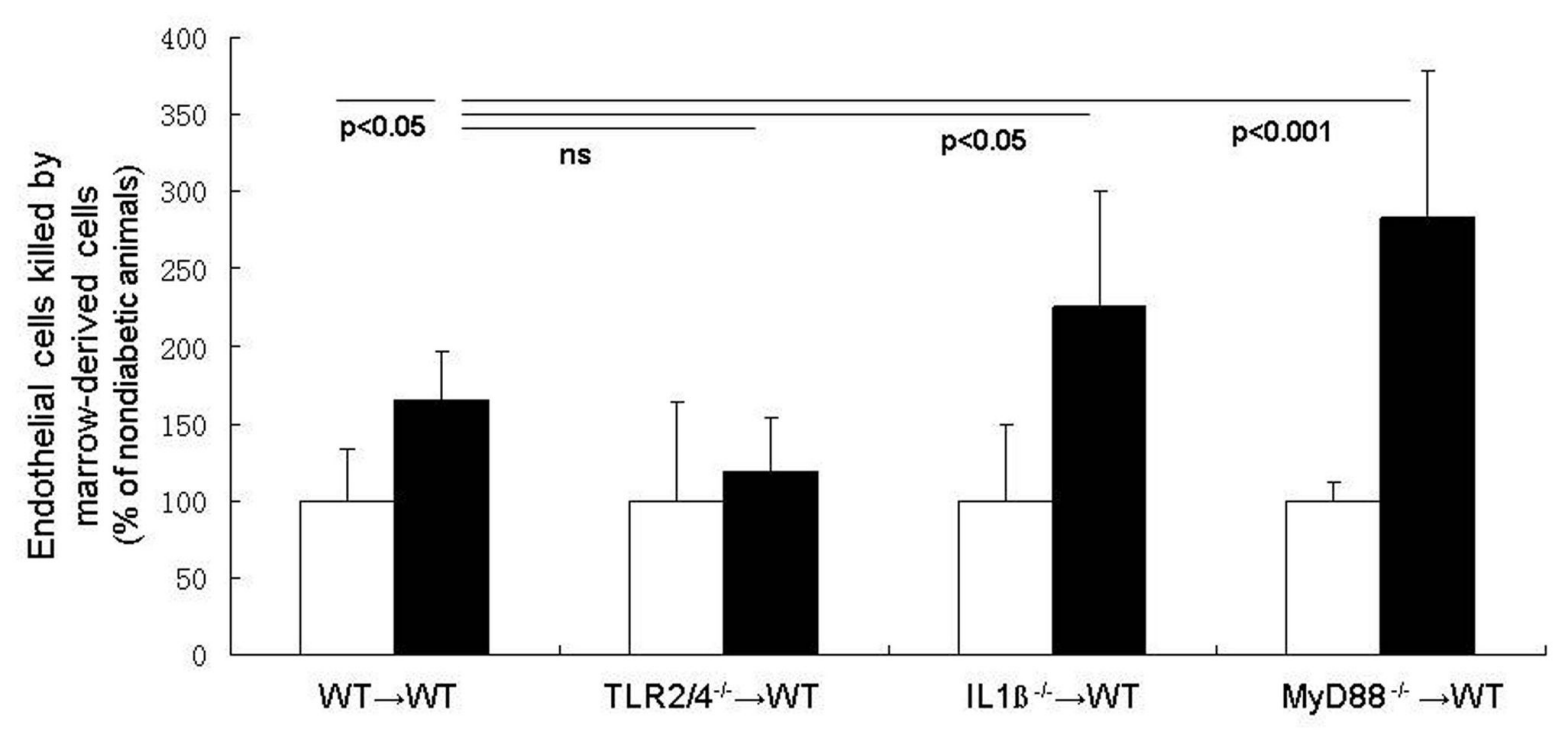
MyD88-dependent pathways in marrow-derived cells exert opposite effects on retinal endothelial cells. Bone marrow cells from diabetic wildtype chemeric mice co-cultured with retinal endothelial cells killed more of the endothelial cells than did marrow cells from wildtype nondiabetic mice. This killing tended to be eliminated if the marrow-derived cells lacked TLR2/4, but the killing was significantly exacerbated if the marrow cells lacked Il-1ßr or MyD88. n=3 per group; representative of two repeat experiments.

Co-culture of bone marrow cells with retinal endothelial cells demonstrate that marrow-derived cells from WT diabetic mice kill more retinal endothelial cells than do comparable cells from nondiabetic animals (Figure 5). Marrow-derived cells from diabetic WT mice increased endothelial death by 74% above that detected when using leukocytes from nondiabetic WT animals. Leukocytes from animals deficient in TLR2/4 caused essentially none of the expected diabetes-induced increase in endothelial death, suggesting that pathways regulated by TLR-2 or -4 (or both) are largely responsible for the leukocyte-mediated death of endothelial cells. Surprisingly, however, deletion of IL-1βr or MyD88-signaling in leukocytes from diabetic mice resulted in significantly more endothelial death than was observed with leukocytes from diabetic WT mice.

### Vascular permeability

**Figure 6:**
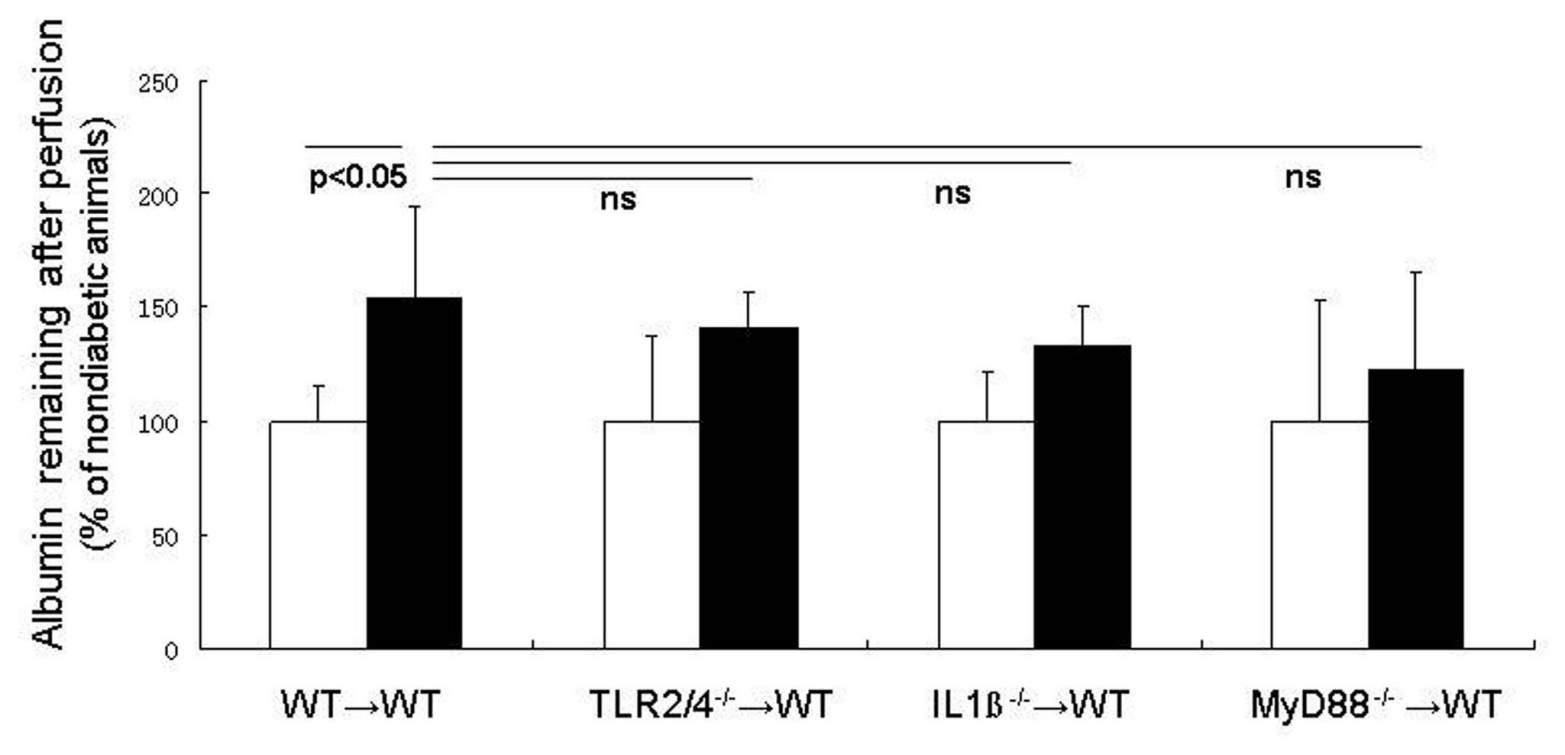
Albumin in neural retina (a possible marker of vascular permeability). The retina wsa perfused to remove blood, and albumin remaining in the retina after that perfusion was interpretd as having leaked into the neural retina. Diabetes significanlty increased that albumin accumulation in wildtpe chimeras. Deletion of IL1β or TLR2/4 or MyD88 from marrow-derived cells had no significant effect on that parameter. n=5 per group.

Increased leakage from the retinal vasculature is a well-recognized consequence of hyperglycemia and diabetes. We perfused anesthetized animals to remove blood from the circulation, and then measured albumin that still remained as an estimate of albumin that leaked into the neural retina. Consistent with expectations, levels of albumin remaining in the retina after perfusion were significantly greater than normal in diabetic WT→WT animals (Figure 6; p<0.05). Deletion of TLR2/4, IL-1βr or MyD88 from the leukocytes partially reduced the amount of albumin that leaked into retina, but none of the results were significantly different from either nondiabetic or diabetic controls. Deletion of MyD88 tended to inhibit albumin accumulation in neural retina more than did deletion of either TLR2/4 or IL-1βr.

### Toll Like Receptor (TLR) activation in diabetic retinas

**Figure 7:**
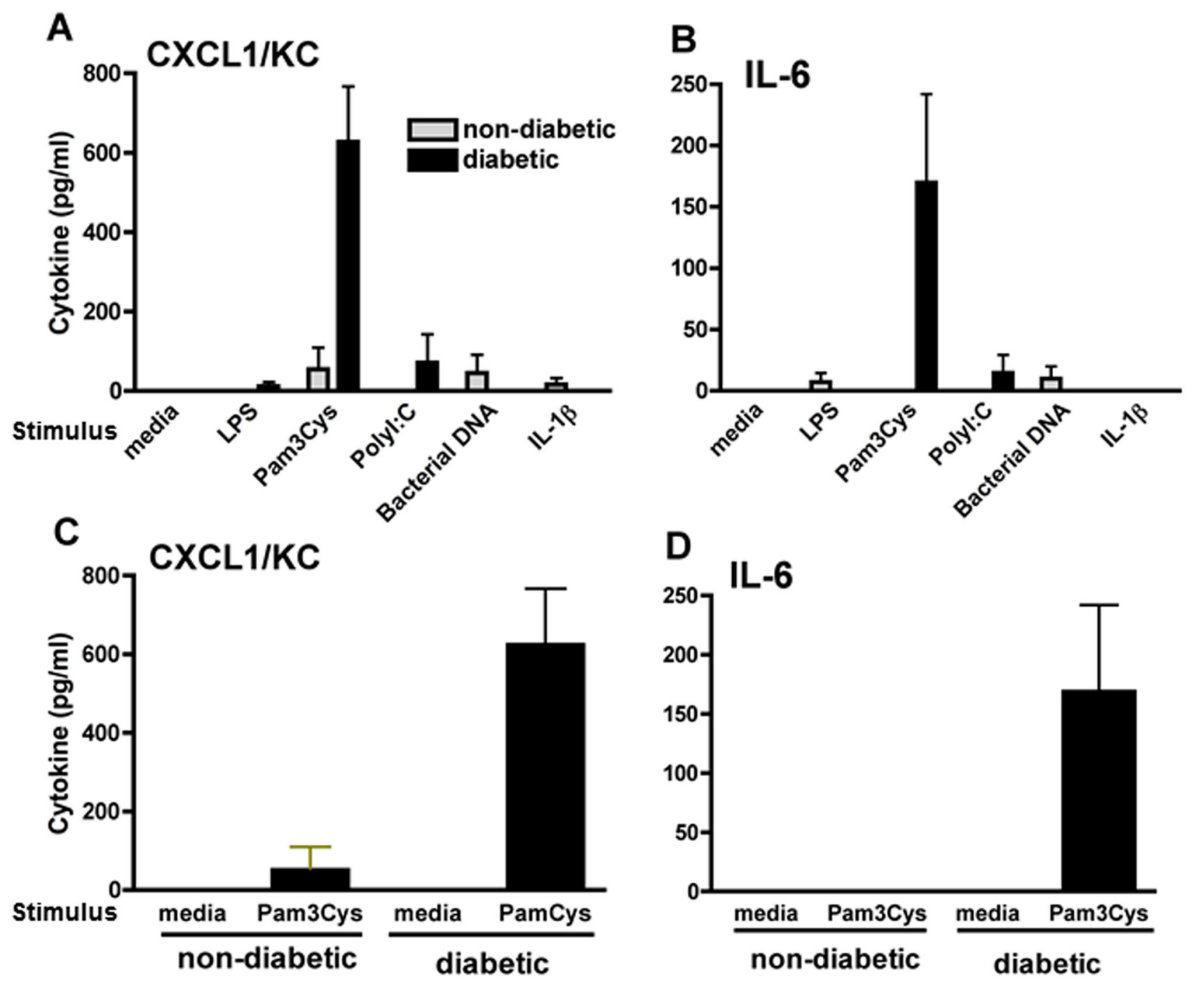
Cytokine production in diabetic retinas. Retinas from diabetic and normal mice were stimulated with TLR ligands, and retinal generation of CXCL1 and IL-6 were examined by ELISA. (A and B) retinas were incubated for 6h with 10 µg/ml of Pam3CysK (to activate TLR2), PolyI:C (TLR3), LPS (TLR4), CpG DNA (TLR9), or with IL-1ß (IL-1r). (C and D) IL-6 and CXCL1 production by retinas following incubation with media alone or containing 10 µg/ml Pam3CysK. Data are mean ± SD of three retinas per group, and are representative of two repeat experiments.

To determine if TLRs are activated in the retina, retinas from C57BL/6 mice were incubated for 6h with 10 µg/ml (final) Pam3CysK (TLR2), PolyI:C (TLR3), LPS (TLR4), and CpG DNA (TLR9), or with IL-1ß that activates IL-1ßr, and cytokine production was measured by ELISA. As shown in Figure 7, IL-6 and CXCL1 were detected in retinas from diabetic, but not normal mice following incubation with Pam3CysK/TLR2. Incubation with other TLR ligands or IL-1ß did not induce cytokine production in either normal or diabetic retinas.

## Discussion

Diabetes causes a number of metabolic and physiologic abnormalities in the retina, but which of these abnormalities contribute to diabetic retinopathy is still under investigation. Many of the molecular and physiologic abnormalities that have been found to develop in the retina in diabetes are consistent with inflammation. Moreover, a number of pharmacologic therapies or genetic modifications that have anti-inflammatory actions significantly inhibit development of different aspects of diabetic retinopathy in animal models [1]. Thus, local inflammatory-like changes induced by diabetes have been implicated in the pathogenesis of diabetic retinopathy.

Inflammation is a nonspecific response to injury that includes a variety of functional and molecular mediators, including recruitment and/or activation of leukocytes, and such leukocytes play a critical role in the development of at least the vascular lesions of diabetic retinopathy. Leukocyte interactions with retinal endothelial cells seems critical to the diabetes-induced capillary degeneration, since deletion of either ICAM or CD18 inhibit the vascular histopathology [17]. During the interaction of these two cell types, release of small lipids (such as leukotrienes and prostanoids) also plays a critical role in the capillary degeneration, because deletion of 5-lipoxygenase from marrow-derived cells only likewise inhibits the diabetes-induced degeneration of retinal capillaries [6]. Diabetic mice lacking iNOS or PARP1 (poly(ADP-ribosyl) polymerase) in their bone marrow-derived cells, likewise were protected against development of the diabetes-induced oxidative stress, induction of inflammatory proteins, and degeneration of retinal capillaries [5]. Depletion of neutrophils in vivo or in vitro inhibited endothelial death otherwise observed diabetes [5]. Since leukocytes play a major role in inflammatory conditions, we sought to investigate the extent to which the leukocytes themselves regulate the local inflammatory response that develops in the retina in diabetes.

MyD88 is a cytosolic adapter protein that plays a central role in the innate and adaptive immune response [8]. In the innate immune system, NF-ĸB activation and inflammation are regulated by MyD88-dependent and independent pathways [8]. MyD88 functions as an essential signal transducer of both the interleukin-1 and TLR signaling pathways which regulate that activation of numerous proinflammatory genes. We found that diabetic, but not normal retinas, were stimulated by Pam3CysK, which activates the TLR2/MyD88 pathway, and supports the concept that this pathway is up-regulated in the retina in diabetes. A variety of physiologic abnormalities that have been implicated in the pathogenesis of diabetic retinopathy failed to develop in retinas of diabetics lacking TLR2/4, further implicating the TLR/MyD88 system in the pathogenesis of diabetic retinopathy.

Removing either MyD88 or the signaling pathways regulated by it (TLRs and IL-1ß) from bone marrow-derived cells significantly inhibited diabetes-induced defects in the retina, including leukostasis, ICAM-1 expression on the luminal surface of the vascular endothelium, retinal superoxide generation, and leukocyte-mediated killing of endothelial cells. This demonstrates that MyD88 is upstream of many of the pro-inflammatory proteins that we and others have demonstrated are important for development of early stages of the retinopathy, and it controls the induction of those pro-inflammatory proteins. In at least one case, however, we found evidence that MyD88-dependent pathways actually antagonized each other, partially inhibiting (TLR2/4^-/-^ leukocytes) or significantly exacerbating (IL1ß^-/-^ or MyD88^-/-^ leukocytes) killing of endothelial cells by leukocytes. This suggests that inhibition of leukocyte mediated killing of endothelial cells in animals lacking TLR2/4 is mediated via a MyD88-independent pathway, but clearly indicates that MyD88 is a factor in diabetes-induced leukocyte activation. If inhibition of MyD88 is to be considered as a therapy for diabetic retinopathy, the balance of these multiple effects of MyD88 will need to be further evaluated.

These studies provide additional evidence that leukocytes play a major role in the local retinal inflammatory response that develops in the retina in diabetes. It is important to note that changes in the marrow-derived cells had a strong influence on the retina itself, causing induction of ICAM-1 in the retinal vascular endothelium and increasing superoxide generation by the whole retina. Thus, it seems likely that MyD88-dependent pathways within circulating white blood cells regulate important aspects of the diabetes-induced local inflammatory response and oxidative stress within the retina. However, MyD88-dependent pathways were less clearly involved in diabetes-induced alterations in albumin accumulation in the retina or leukocyte-mediated killing of endothelial cells. Additional studies will be required to more fully assess the suitability of targeting of MyD88 to inhibit diabetic retinopathy.

Cells circulating in the blood might affect retinal endothelial cells and retina by either direct contact or via release of soluble factors which damage the endothelium. Studies have shown that marrow-derived cells can generate leukotrienes or similar prostenoids, and that transcellular delivery of prostanoid precursors from blood-borne cells to the retina can contribute to the death of endothelial cells and the pro-inflammatory state in diabetic retinopathy [6,18]. In addition, release of cytokines and reactive molecules from activated leukocytes are well known to exert effects on the nearby vasculature.

The retinal abnormalities caused by diabetes and studied herein were selected because of their relevance to the pathogenesis and progression of diabetic retinopathy. Since most of them were shown to be MyD88-dependent, we thus postulate that MyD88-dependent pathways (and presumably the innate immune system) are important in the development of metabolic and physiologic abnormalities that contribute to diabetic retinopathy. Importantly, the studies also demonstrate that bone marrow-derived cells drive the local inflammatory response that develops in the retina (and presumably other tissues), in that MyD88-regulated pathways within the circulating marrow-derived cells regulated endothelial expression of ICAM-1 and supperoxide generation by the retina in diabetes. We postulate that MyD88 is novel target to inhibit early abnormalities of diabetic retinopathy and perhaps other complications of diabetes.

## Conclusions

MyD88-dependent pathways play an important role in the development of diabetes-induced inflammation in the retina, and inhibition of MyD88 might be a novel target to inhibit early abnormalities of diabetic retinopathy and perhaps other complications of diabetes.

## References

[1] Tang J, Kern TS (2011) Inflammation in diabetic retinopathy. Prog Retin Eye Res 30: 343-358. doi:10.1016/j.preteyeres.2011.05.002. PubMed: 21635964. 21635964 10.1016/j.preteyeres.2011.05.002 PMC3433044

[2] Kern TS (2007) Contributions of inflammatory processes to the development of the early stages of diabetic retinopathy. Exp Diabetes Res: 95103: 95103 PubMed: 18274606. 10.1155/2007/95103 PMC2216058 18274606

[3] Adamis AP, Berman AJ (2008) Immunological mechanisms in the pathogenesis of diabetic retinopathy. Semin Immunopathol 30: 65-84. doi:10.1007/s00281-008-0111-x. PubMed: 18340447. 18340447 10.1007/s00281-008-0111-x

[4] Kaul K, Hodgkinson A, Tarr J, Kohner EM, Chibber R (2010) Is Inflammation a Common Retinal-Renal-Nerve Pathogenic Link in Diabetes? Curr Diabetes Rev. 10.2174/157339910793360851 20594163

[5] Li G, Veenstra AA, Talahalli RR, Wang X, Gubitosi-Klug RA et al. (2012) Marrow-Derived Cells Regulate the Development of Early Diabetic Retinopathy and Tactile Allodynia in Mice. Diabetes, 61: 3294–303. PubMed: 22923475. 22923475 10.2337/db11-1249 PMC3501859

[6] Talahalli R, Zarini S, Tang J, Li G, Murphy R et al. (2012) Leukocytes regulate retinal capillary degeneration in the diabetic mouse via generation of leukotrienes. J Leukoc Biol, 93: 135–43. PubMed: 23108096. 23108096 10.1189/jlb.0112025 PMC3525833

[7] Tang D, Kang R, Coyne CB, Zeh HJ, Lotze MT (2012) PAMPs and DAMPs: signal 0s that spur autophagy and immunity. Immunol Rev 249: 158-175. 22889221 10.1111/j.1600-065X.2012.01146.x PMC3662247

[8] Kawai T, Akira S (2007) Signaling to NF-kappaB by Toll-like receptors. Trends Mol Med 13: 460-469. doi:10.1016/j.molmed.2007.09.002. PubMed: 18029230. 18029230 10.1016/j.molmed.2007.09.002

[9] Li G, Tang J, Du Y, Lee CA, Kern TS (2011) Beneficial effects of RAGE-Ig fusion protein on early diabetic retinopathy and tactile allodynia. Mol Vis 17: 3156-3165. PubMed: 22171162. 22171162 PMC3235538

[10] Gubitosi-Klug RA, Talahalli R, Du Y, Nadler JL, Kern TS (2008) 5-Lipoxygenase, but not 12/15-Lipoxygenase, Contributes to Degeneration of Retinal Capillaries in a Mouse Model of Diabetic Retinopathy. Diabetes 57: 1387-1393. doi:10.2337/db07-1217. PubMed: 18346986. 18346986 10.2337/db07-1217 PMC4444435

[11] Zheng L, Du Y, Miller C, Gubitosi-Klug RA, Ball S et al. (2007) Critical role of inducible nitric oxide synthase in degeneration of retinal capillaries in mice with streptozotocin-induced diabetes. Diabetologia 50: 1987-1996. doi:10.1007/s00125-007-0734-9. PubMed: 17583794. 17583794 10.1007/s00125-007-0734-9

[12] Du Y, Miller CM, Kern TS (2003) Hyperglycemia increases mitochondrial superoxide in retina and retinal cells. Free Radic Biol Med 35: 1491-1499. doi:10.1016/j.freeradbiomed.2003.08.018. PubMed: 14642397. 14642397 10.1016/j.freeradbiomed.2003.08.018

[13] Su X, Sorenson CM, Sheibani N (2003) Isolation and characterization of murine retinal endothelial cells. Mol Vis 9: 171-178. PubMed: 12740568. 12740568

[14] Kern TS, Miller CM, Du Y, Zheng L, Mohr S et al. (2007) Topical administration of nepafenac inhibits diabetes-induced retinal microvascular disease and underlying abnormalities of retinal metabolism and physiology. Diabetes 56: 373-379. doi:10.2337/db05-1621. PubMed: 17259381. 17259381 10.2337/db05-1621

[15] Du Y, Tang J, Li G, Berti-Mattera L, Lee CA et al. (2010) Effects of p38 MAPK inhibition on early stages of diabetic retinopathy and sensory nerve function. Invest Ophthalmol Vis Sci 51: 2158-2164. doi:10.1167/iovs.09-3674. PubMed: 20071676. 20071676 10.1167/iovs.09-3674 PMC2868413

[16] Kern TS, Miller CM, Tang J, Du Y, Ball SL et al. (2010) Comparison of three strains of diabetic rats with respect to the rate at which retinopathy and tactile allodynia develop. Mol Vis 16: 1629-1639. PubMed: 20806092. 20806092 PMC2927372

[17] Joussen AM, Poulaki V, Le ML, Koizumi K, Esser C et al. (2004) A central role for inflammation in the pathogenesis of diabetic retinopathy. Faseb J 18: 1450-1452. PubMed: 15231732. 15231732 10.1096/fj.03-1476fje

[18] Talahalli R, Zarini S, Sheibani N, Murphy RC, Gubitosi-Klug RA (2010) Increased synthesis of leukotrienes in the mouse model of diabetic retinopathy. Invest Ophthalmol Vis Sci 51: 1699-1708. doi:10.1167/iovs.09-3557. PubMed: 19834040. 19834040 10.1167/iovs.09-3557 PMC2868429

